# Delayed Treatment with Systemic (S)-Roscovitine Provides Neuroprotection and Inhibits *In Vivo* CDK5 Activity Increase in Animal Stroke Models

**DOI:** 10.1371/journal.pone.0012117

**Published:** 2010-08-12

**Authors:** Bénédicte Menn, Stéphane Bach, Teri L. Blevins, Mark Campbell, Laurent Meijer, Serge Timsit

**Affiliations:** 1 Neurokin S.A., Institut de Neurobiologie de la Méditerranée, Marseille, France; 2 USR3151, CNRS, Station Biologique de Roscoff, Roscoff, France; 3 Efficacy Pharmacology, MDS Pharma Services, Bothell, Washington, United States of America; 4 Département de Neurologie, CHRU Brest, Faculté de Médecine et des Sciences de la Santé de Brest, Brest, France; INSERM U901, France

## Abstract

**Background:**

Although quite challenging, neuroprotective therapies in ischemic stroke remain an interesting strategy to counter mechanisms of ischemic injury and reduce brain tissue damage. Among potential neuroprotective drug, cyclin-dependent kinases (CDK) inhibitors represent interesting therapeutic candidates. Increasing evidence indisputably links cell cycle CDKs and CDK5 to the pathogenesis of stroke. Although recent studies have demonstrated promising neuroprotective efficacies of pharmacological CDK inhibitors in related animal models, none of them were however clinically relevant to human treatment.

**Methodology/Principal Findings:**

In the present study, we report that systemic delivery of (S)-roscovitine, a well known inhibitor of mitotic CDKs and CDK5, was neuroprotective in a dose-dependent manner in two models of focal ischemia, as recommended by STAIR guidelines. We show that (S)-roscovitine was able to cross the blood brain barrier. (S)-roscovitine significant *in vivo* positive effect remained when the compound was systemically administered 2 hrs after the insult. Moreover, we validate one of (S)-roscovitine *in vivo* target after ischemia. Cerebral increase of CDK5/p25 activity was observed 3 hrs after the insult and prevented by systemic (S)-roscovitine administration. Our results show therefore that roscovitine protects *in vivo* neurons possibly through CDK5 dependent mechanisms.

**Conclusions/Significance:**

Altogether, our data bring new evidences for the further development of pharmacological CDK inhibitors in stroke therapy.

## Introduction

Despite numerous clinical trials, neuroprotective therapies in ischemic stroke have failed in human [1], [2]. Protecting the brain tissue from injury remains however an interesting, although quite challenging, option in stroke treatment strategies [3]. It is indeed widely accepted that not all brain cells die immediately after the insult. Surrounding a core of severe and rapid tissue injury, brain cell death spreads more slowly in a heterogeneous region called the penumbra that could still be salvaged [4]. Numerous preclinical studies have therefore shown that it is possible to achieve significant reductions of ischemic injury using neuroprotective strategies [5] but they failed later in clinical trials. Reasons for the unsuccessful translation of neuroprotective therapies from animal to human are probably multiple [6]. This has led the Stroke Academic Industry Roundtable (STAIR) to make recommendations to improve the quality of preclinical studies of purported acute stroke therapies [7], [8]. One aspect concerns the preclinical stage of the drug development where insufficient dose-response or time-window studies, inappropriate drug delivery protocol, or brain penetration issues are often encountered. *In vivo* analysis of the mechanism targeted by the drug is also among the aspects that should be improved.

Because excitotoxicity is a pivotal mechanism in ischemic stroke, most of human trials in neuroprotection have focused on glutamate release and glutamate receptor, but without success [5]. Such approaches targeted only the surface of the neurons. They did not act along the transduction pathways involved in cellular death nor on the extrinsic stressors associated with stroke, such as activation of glial cells or inflammation. It became therefore warranted that the “ideal” neuroprotective drug should display a broad action mode by influencing concomitantly apoptotic, inflammatory and excitotoxic pathways and act not only on neurons, but also on astrocytes and oligodendrocytes.

Among potential neuroprotective drugs, cyclin-dependent kinase (CDK) inhibitors represent interesting candidates to overcome such a challenge. There is now abundant evidence that the family of serine/threonine kinases CDKs have essential functions in the apoptotic and excitotoxic pathways [9]–[11]. Within this cascade of events, CDK5 exerts a central role as a key regulator of neuronal death and survival [11]. CDK5 is associated with cerebral ischemia. CDK5 activity in the brain is triggered by its binding partners p39 and p35 [12]. Deregulation of CDK5 under pathological conditions is induced by calpain- mediated cleavage of p35 into a shorter form p25. The p25 fragment triggers CDK5 hyperactivation and translocation of the p25/CDK5 complex to the cytoplasm where it hyperphosphorylates a number of substrates, leading to neuronal death [13]. Moreover, up-regulation of cell cycle proteins (cyclin D1, CDK4, and CDK2) is associated with neuronal apoptosis, as well as proliferation and activation of glial cells after cerebral ischemia [14]–[17].

Despite accumulating evidence that CDK5 and mitotic CDKs may be essential targets for ischemic stroke, only few attempts to modulate their *in vivo* activity have been reported so far [18]–[23]. Here we follow the STAIR recommendations to investigate the *in vivo* neuroprotective potential of (S)-roscovitine, a well known inhibitor of CDK5 and mitotic CDKs, in acute stroke. We show that systemic delivery of (S)-roscovitine in the appropriate formulation is neuroprotective in models of focal ischemia with an appropriate therapeutic time window and cross the blood brain barrier. We also show that (S)-roscovitine regulates CDK5 *in vivo* activity after stroke, suggesting that CDK5 is involved in (S)-roscovitine *in vivo* beneficial effect on ischemic brain.

## Results

### Systemic (S)-roscovitine protects from cell death in a permanent model of focal cerebral ischemia

Although it has been established that (R)-roscovitine is able to cross the blood brain barrier [24], [25], its neuroprotective effect after a systemic delivery route has never been reported in stroke models. A systemic administration of the drug is moreover the only feasible route of delivery in human stroke therapy. We therefore investigated the beneficial effect of systemic (S)-roscovitine, the levogyre form of roscovitine, in a model of permanent focal ischemia in adult mice. This model consisted of the permanent unilateral occlusion of the distal middle cerebral artery by electrocoagulation (pMCAo; [26] modified from [27]). In this model of distal occlusion, mice exhibit an ischemic lesion that is exclusively ipsilateral and restricted to the temporoparietal cortex [26]. The infarction is observed as early as 30 min after injury and expands outwards to reach a maximum volume 24 hrs after occlusion [26]. Preliminary characterization of the infarcted tissue at 3 hrs after pMCAo using 2, 3, 5 tetrazolium chloride (TTC) staining revealed a gradient of mitochondrial activity in the mouse brain. In addition to the healthy peri-infarct region that displayed a pink strong TTC staining, we observed two infarcted regions of various TTC staining intensity: a white region of severely decreased mitochondrial activity (called “ischemic core” by Guegan and colleagues [26]), and a pinkish region of moderately decreased mitochondrial activity representing a mitochondrial hypometabolic area that cannot be assimilated to penumbra (also called “ischemic zone” [26]) (**Figure S1**).

**Figure 1:**
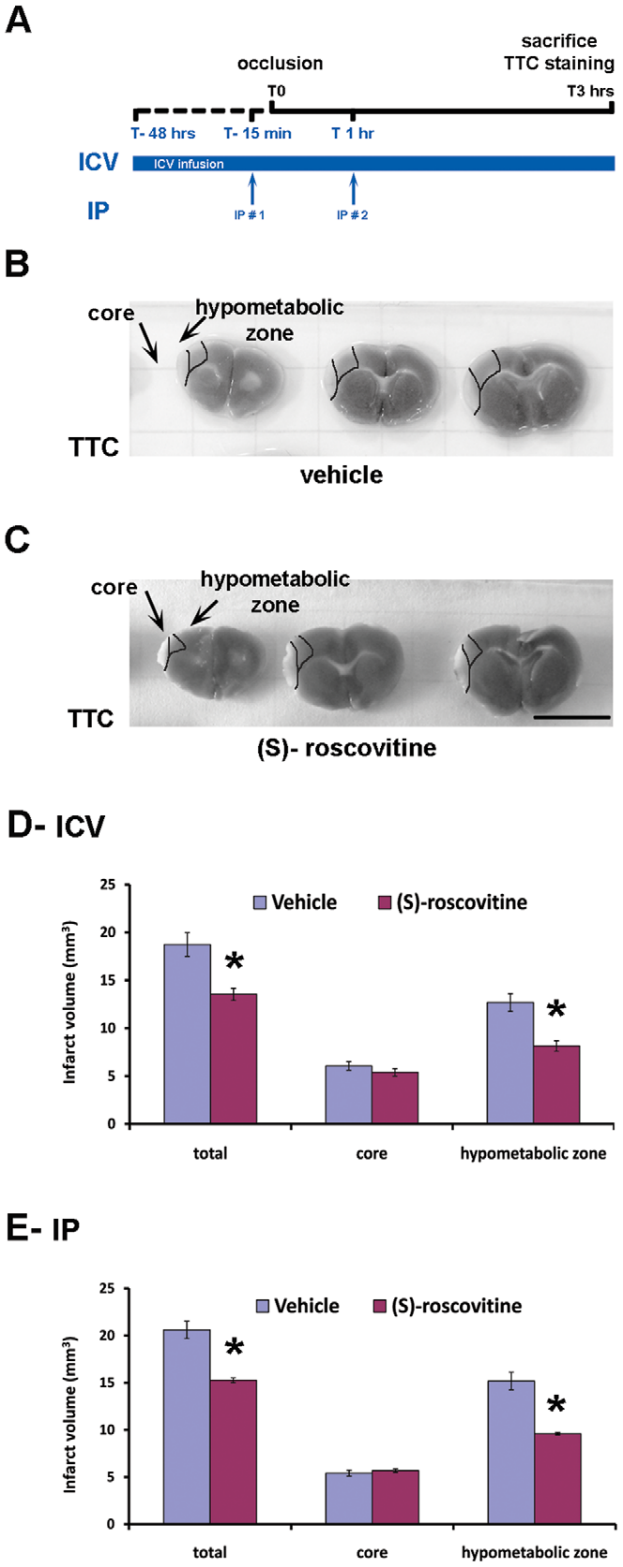
Systemic (S)-roscovitine displays neuroprotective efficacy in an adult mouse model of permanent MCAo. (**A**) Scheme of the experimental protocol. Vehicle or (S)-roscovitine were administered to pMCAo mice either by ICV infusion at a concentration of 500 µM starting 48 hrs before pMCAo or by two intraperitoneal (IP) injections at a dose of 25 mg/kg done 15 min pre- and 1 hr post-MCAo. Animals were sacrificed at 3 hrs after the occlusion and their brains were analyzed by TTC staining. (**B, C**) Bright field photomicrographs of adjacent 1 mm thick coronal brain sections of animals ICV- treated with vehicle- (**B**) and (S)-roscovitine- (**C**) and analyzed after 3 hrs of pMCAo. Ischemic core, hypometabolic zone, and healthy areas were delimited. (**D, E**) Core and hypometabolic zone were identified by TTC staining within the entire brain of vehicle- and (S)-roscovitine- treated animals and their respective volumes were determined using the ImageJ software. Total volume of the infarcted regions was determined as the sum of the core and hypometabolic zone volumes. The corrected volume of the total infarcted region significantly decreased by 27.7% and 30.7% in lesioned animals that received (S)-roscovitine administered respectively ICV (**D**) or IP (**E**) in comparison to those that received vehicle only. Note that, while the volume of the core area is comparable in both vehicle- and (S)-roscovitine- treated animals, the volume of the hypometabolic zone shows, compared to the vehicle- treated animals, a 35.8% and 38.9% decrease respectively in animals that received (S)-roscovitine administered ICV (**D**) or IP (**E**). Scale bar: A, B: 9.5 mm * p<0.01, *t*-test.

Using this model, we evaluated the neuroprotective effect of systemic (S)-roscovitine on the ischemic volume in adult mice brains 3 hrs after the onset of pMCAo (**Figure 1A**). Two experiments using different delivery protocols of the compound were performed. In the first set of experiment, the compound or its vehicle control (n = 5 mice per group) was first administered to the animal intracerebroventricularly (ICV), via a micro-osmotic pump, 48 hrs before the ischemic insult and throughout the duration of the pMCAo, in order to validate (S)-roscovitine beneficial effect. In the second set of experiment, the compound or its vehicle control (n = 5 mice per group) was delivered using 2 successive systemic (IP) injections at 15 min prior and 1 hr after the occlusion. In this last experiment, animal body temperature and blood glucose concentration were recorded during the surgical procedure. No significant differences in these physiological parameters were observed between the 2 experimental groups in this experiment (**Table S1**). Hypothermia was observed for both groups at 3 hrs post-occlusion despite heating procedures. No significant difference was however observed between the 2 groups.

Ischemic core, ischemic hypometabolic zone and healthy area were identified based on TTC- stained brain sections of vehicle- (**Figure 1B**) and (S)-roscovitine- (**Figure 1C**) treated animals. Analysis of the volume of each area revealed that (S)-roscovitine treatment led to a 28% decrease of the total infarct volume compared to vehicle-treated animals (**Figure 1D**). While similar infarct volumes were measured in the ischemic core area both in vehicle- and (S)-roscovitine- treated animals, a significant 36% reduction of the ischemic zone volume was observed for the animals treated with (S)-roscovitine in comparison to those which received the vehicle only (**Figure 1D**). Similar observations were made after systemic administration of (S)-roscovitine to the animals with a 31% decrease of the total infarct volume (**Figure 1E**). In this case, the volume of the ischemic zone was significantly decreased by 39% in (S)-roscovitine- treated animals compared to vehicle treated- animals (**Figure 1E**).

### Development of injectable formulation for (S)-roscovitine

In order to establish a drug delivery protocol that is suitable for human patient, we developed an injectable formulation for (S)-roscovitine. Because of its low solubility at neutral pH (**Table 1**), we screened for excipients that could increase (S)-roscovitine solubility. Excipients selected in this study were surfactants commercially available for human formulation [28]. Among the formulations tested, we found that 30% hydroxypropyl β-cyclodextrin (HPbCD) allowed the maximum solubilization of the compound (**Table 1**). Blood brain barrier permeability of (S)-roscovitine was therefore verified using this formulation. Our data showed that exposure of the brain to (S)-roscovitine was approximately 15% of that found in plasma of adult rat (**Table 2**).

**Table 1:** Formulation development for injectable (S)-roscovitine.

Vehicle	Solubility (mg/mL)
Buffer pH 3	1.2
Buffer pH 7.4	0.02
Cremophor EL 5% pH 7.4	1.1
Tween 80 5% pH 7.4	1.0
Hydroxypropyl β-cyclodextrine 10% pH 7.4	0.9
Hydroxypropyl β-cyclodextrine 30% pH 7.4	4.4

Because of the low solubility of (S)-roscovitine at neutral pH, several excipients were screened in order to increase its solubilization. Excipients presented in this table are surfactants commercially available for human injectable formulation.

**Table 2:** Blood brain barrier (BBB) permeability of injectable (S)-roscovitine.

Compound	Route	Dose (mg/kg)	Sampling time	Brain/Plasma
(S)-roscovitine	i.v.	20	15 min	0.15 +/− 0.05
	i.v.	30	15 min	0.17 +/− 0.07

(S)-roscovitine permeability to the BBB solubilized in 4% HPbCD was examined after IV administration of the compound to healthy adult rats. (S)-roscovitine was administered at 2 doses (20 and 30 mg/kg) to the animals (n = 2 rats/dose) and (S)-roscovitine level was determined in the brain and plasma 15 min later. Approximately 15% of the IV injected (S)-roscovitine was able to cross the BBB in healthy adult rats.

### Delayed treatment with systemic (S)-roscovitine provides neuroprotection after transient focal cerebral ischemia

**Figure 2:**
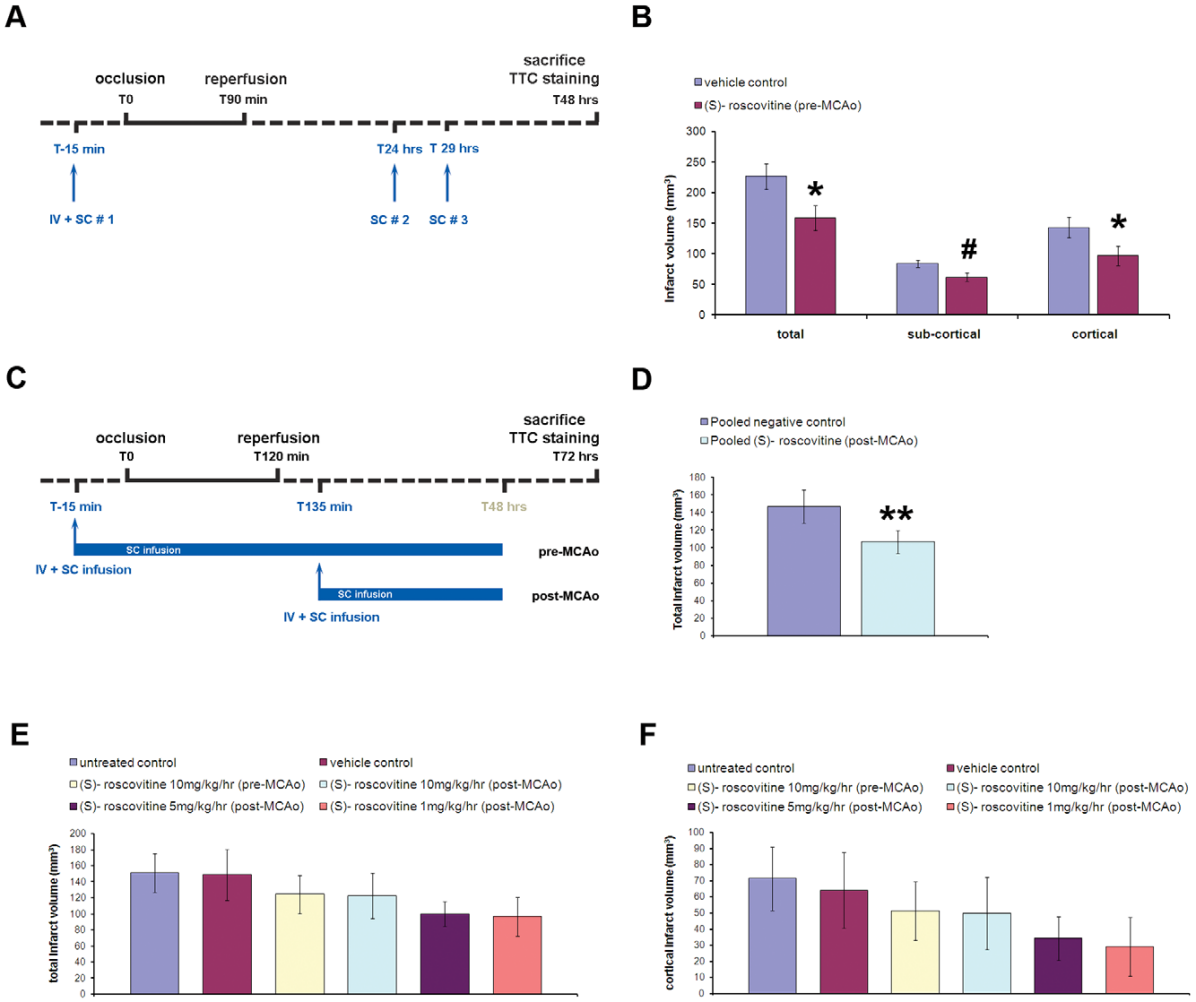
Systemic (S)-roscovitine decreases lesion infarct size after transient focal ischemia in rat when administered prior or 2h15 after the ischemia onset. Two independent blinded studies were performed at Neurokin (**A, B**) and by MDS Pharma Services (**C–F**). (**A**) Scheme of the experimental protocol performed at Neurokin. Transient focal ischemia was induced in adult rats for 90 min. (S)-roscovitine (n = 16 rats) or its vehicle control (n = 18 rats) was administered 15 min prior the occlusion by a IV bolus at one dose (25 mg/kg) and 3 successive SC injections at one dose (54 mg/kg) performed 15 min prior, 24 and 29 hrs after the occlusion. Rats were analyzed by TTC staining at 48 hrs post-occlusion. (**B**) Evaluation of the corrected infarct volumes in this experiment revealed a significant reduction by 30%, 33% and 26% respectively of the total, sub-cortical, and cortical areas of the brain lesion in presence of (S)-roscovitine administered 15 min prior the ischemia onset. (**C**) Scheme of the experimental protocol performed at MDS PS. tMCAo was induced for 120 min. (S)-roscovitine (3 doses tested, n = 12 rats per dose) or its vehicle control (n = 13 rats) was administered to tMCAo rats either prior (pre-MCAo) or 135 min after (post-MCAo) the ischemia onset. (S)-roscovitine or vehicle administration was done by an IV bolus at one dose (25 mg/kg) followed by SC infusion at 3 decreasing doses for 48 hrs (10, 5, and 1 mg/kg). In addition, untreated control tMCAo animals (n = 11 rats) were also prepared. Animals were sacrificed at 72 hrs after the occlusion and their brains were analyzed by TTC staining. (**D**) Analysis of the corrected infarct volumes at 72 hrs after a 120 min tMCAo showed no significant difference in total infarct volume between the vehicle control group and the untreated control group, indicating the absence of effect of the vehicle used in our study. Therefore, both untreated and vehicle treated groups were pooled in one negative control group. Similarly, (S)-roscovitine- treated groups were pooled in one group containing the three post-MCAo treated groups only (post- MCAo). A significant decrease of the corrected total infarct volumes was observed when the pooled negative control was compared to the pooled (S)-roscovitine post-MCAo treated groups. (**E**) In contrast, no significant difference was observed between the control groups and each (S)-roscovitine treated group independently of the dose or the administration time. (**F**) Although not statistically significant, a dose-dependent beneficial effect of (S)-roscovitine on the lesion volume was observed mostly on the cortical region of the brain lesion by reducing the volume by 30%, 52% and 58% respectively at 10, 5 and 1 mg/kg/hr, *p<0.01, ** p<0.05, *t*-test #p<0.01, Mann Whitney test.

STAIR recommendations strongly suggest establishing a drug efficacy both in permanent and transient models of acute stroke and at least in 2 rodent species [7], [8]. We therefore evaluated (S)-roscovitine neuroprotective efficacy in a rat model of transient focal ischemia that mimics more closely what happens in human stroke, where recanalization occurs frequently. Moreover, in order to validate the reproducibility of our data [8], 2 independent blinded studies were therefore performed both at Neurokin and MDS Pharma Services facilities using the model of transient occlusion of the middle cerebral artery (tMCAo). In our first study, transient focal ischemia was induced for 90 min in rats (**Figure 2A**). (S)-roscovitine (n = 16 rats) or its vehicle control (n = 18 rats) was administered by IV bolus at 15 min prior to ischemia and by 3 successive SC injections performed at 15 min prior and 24 and 29 hrs after the occlusion. Animals were analyzed at 48 hrs post-tMCAo by TTC staining. Animal body temperature and blood glucose concentration were recorded during the surgical procedures. A significantly lower temperature was observed at the post-reperfusion time for the (S)-roscovitine (pre-MCAo) group compared to the control one. Moreover, a higher blood glucose concentration was observed at the post-reperfusion time for the (S)-roscovitine group compared to the control one (**Table S2**). Overall animal mortality in this study was 3%. No significant difference of animal mortality was observed between the 2 experimental groups (p = 0.31, Fisher exact test). Analysis of the infarct volume at 48 hrs post-occlusion showed that (S)-roscovitine significantly decreased by 30%, 33%, and 26% respectively the total, cortical and sub-cortical volumes of the infarcted regions in rats after 90 min of tMCAo (**Figure 2B**).

In our second set of experiments, tMCAo was induced for 120 min in rats (**Figure 2C**). (S)-roscovitine (n = 11–12 rats) or its vehicle control (n = 13 rats) was administered by a IV bolus immediately followed by continuous systemic (SC) infusion performed either 15 min before (pre-MCA group) or 135 min after (post-MCA groups) the ischemia onset were tested using of the compound. In this last condition, various decreasing doses of (S)-roscovitine were also tested. Animal body temperature and blood glucose concentration were recorded during the surgical procedures. If no significant differences in these physiological parameters were observed for the animals that received the compound after the ischemia onset (post-MCA groups), a higher blood glucose concentration was observed at the post-reperfusion time for the (S)-roscovitine group compared to the control one in the pre-MCAO group (**Table S3**). Overall animal mortality in this study was 3%. No significant difference of animal mortality was observed between the different experimental groups (p = 0.51–0.54, Fisher exact test). In this set of experiment, a slight difference, although no significant, of the lesion volume was observed between controls (pooled untreated and vehicle control groups) and (S)-roscovitine pre-treated group using high dose (**Figure 2E**). Furthermore, a significant decrease of 27% in the total infarct volume was observed between post-treated groups (pooled (S)-roscovitine post-MCA group) and controls (**Figure 2D**). Although not significantly different, our data also showed that lower doses of (S)-roscovitine (5 and 1 mg/kg/hr) tend to exhibit a stronger neuroprotective effect that the highest dose (10 mg/kg/hr) (**Figure 2E**). (S)-roscovitine dose-dependent effect was mainly observed in the cortical area of the brain lesion, where the volume reductions were of 52% and 59% for the two lowest doses (**Figure 2F**).

### CDKs are involved in (S)-roscovitine mediated neuroprotective effects

**Figure 3:**
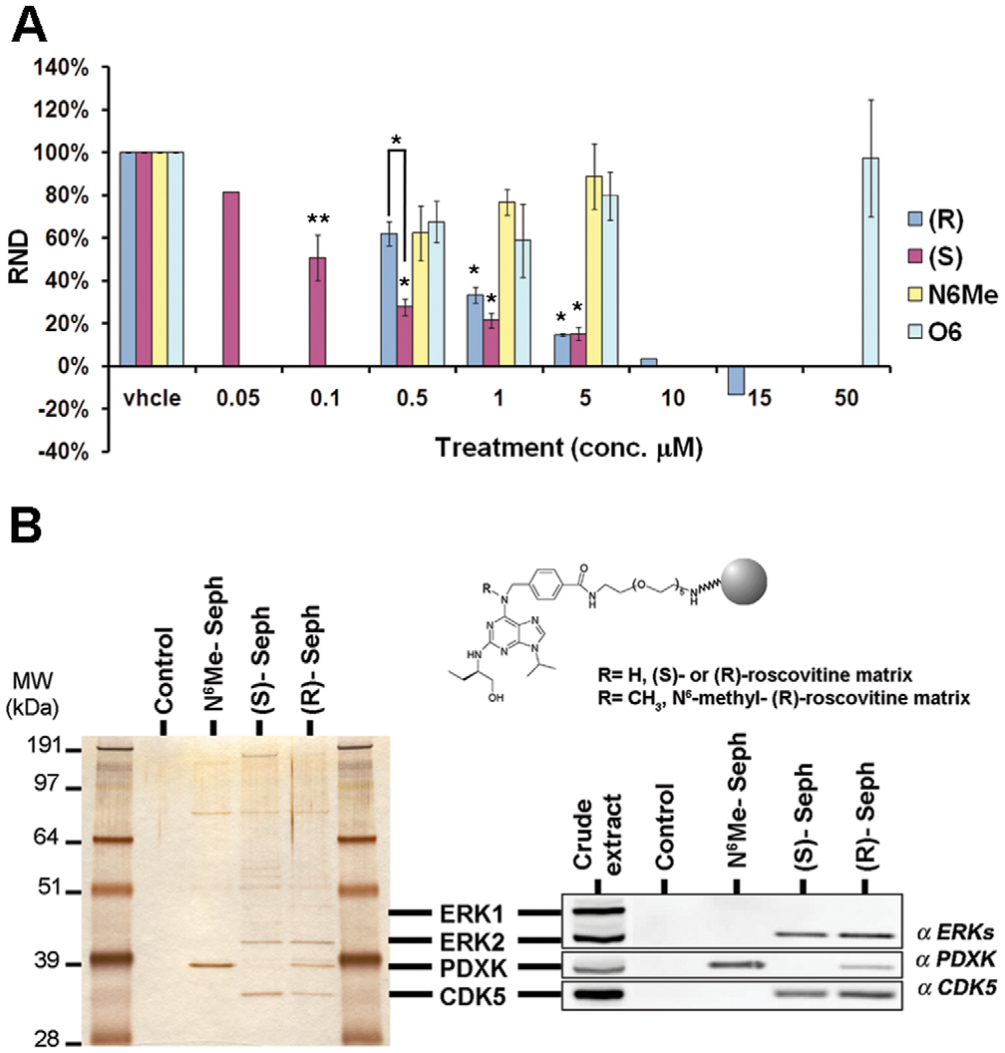
(S)-roscovitine neuroprotective effects are at least in part mediated by CDKs. (**A**) Neuroprotective effects of roscovitine analogs were assessed using an *in vitro* model of excitotoxicity. Mixed hippocampal cultures were grown for 10 div and exposed to KA (200 µM) for 5 hrs and to roscovitine stereoisomers or kinase inactive analogs added at the same time as the toxic agent. Beneficial effects of roscovitine on KA-induced neuronal death were assessed by phase contrast observation and PI-staining. Relative neuronal death (RND) following KA treatment was measured at different concentrations of (R)-roscovitine [vhcle (n total = 322 neurons), 0.5 (n = 222), 1 (n = 398), 5 (n = 392), 10 (n = 80), and 15 (n = 83) µM], (S)-roscovitine [vhcle (n = 766), 0.05 (n = 76), 0.1 (n = 272), 0.5 (n = 511), 1 (n = 528), and 5 (n = 479) µM], N^6^Me-roscovitine [vhcle (n = 326), 0.5 (n = 199), 1 (n = 163), and 5 (n = 173) µM], and O^6^-roscovitine [vhcle (n = 391), 0.5 (n = 174), 1 (n = 180), 5 (n = 240), and 50 (n = 75) µM]. While both (R) and (S) stereoisomers displayed significant dose-dependent beneficial effect on KA-induced neuronal death, the kinase- inactive roscovitine analogs, N^6^-methyl-(R)-roscovitine and O^6^-(R)-roscovitine, did not exhibited any neuroprotective effect *in vitro*. Note the significantly increased neuroprotective activity of (S)-roscovitine compared to the (R) stereoisomer at 0.5 µM. (**B**) Affinity chromatography purification of roscovitine targets in the adult rat brain. Total brain extract (500 µg) of one healthy mouse was loaded on immobilized (S)-, (R)-, and N^6^-methyl-(R)-roscovitine (N6Me), as well as on ethanolamine beads (control without inhibitor). The beads were extensively washed and the bound proteins analyzed by SDS-PAGE followed by silver staining (*left*) and Western blotting (*right*) against Erk1/2, PDXK, and CDK5. Note that, as previously described in [30], [31], only N^6^-methyl-(R)-roscovitine and (R)-roscovitine bind pyridoxal kinase (PDXK) whereas Erk2 and CDK5 bind to both (S) and (R) stereoisomers of roscovitine but not to N^6^-methyl-(R)-roscovitine. * p<0.01 ** p<0.05, *t*-test.

In order to identify (S)-roscovitine targets after stroke, we first used a model of selective neuronal excitotoxicity using mixed neuron/glia hippocampal cell cultures and the non- NMDA agonist kainate (KA) (**Figure S2**). In the adult rat brain, KA leads mainly to neuronal death with apoptotic features [29] evocative of what is generally observed in the penumbra region after ischemic injury. The neuroprotective effects on KA-induced neuronal death were evaluated following exposure to the (R) and (S) stereoisomers of roscovitine, as well as N^6^-methyl (R)-roscovitine and O^6^-(R)-roscovitine that are two compounds designed for their inability to inhibit CDKs [30] (**Figure 3A**). (R)- and (S)-roscovitine displayed a significant dose-dependent neuroprotective effect on KA- induced excitotoxicity (**Figure 3A**). We also noticed that the (S)-roscovitine displayed a significantly stronger neuroprotective effect than the (R) stereoisomer at the 0.5 µM dose in our culture system (**Figure 3A**). In contrast, both the kinase inactive N^6^-methyl (R)-roscovitine and O^6^-(R)-roscovitine showed no significant neuroprotective effect even at high concentrations (**Figure 3A**). In the adult mouse brain, we used an affinity chromatography approach, based on roscovitine immobilized on a solid matrix as previously described [9], [30]. Both (S) and (R) stereoisomers, as well as N^6^-methyl-(R)-roscovitine, were immobilized on sepharose beads (**Figure 3B**). Analysis of the bound proteins by SDS-PAGE followed by silver staining and Western blotting showed that (S)-roscovitine preferentially bound Erk2 and CDK5 in the adult mouse brain (**Figure 3B**). Moreover, immobilized (S)- isomer did not bind PDXK as previously described [30], [31], in contrast to immobilized (R)-roscovitine and N^6^-methyl-(R)-roscovitine (**Figure 3B**).

### CDK5 activity is enhanced following focal cerebral ischemia and constitutes an *in vivo* target of (S)-roscovitine

**Figure 4:**
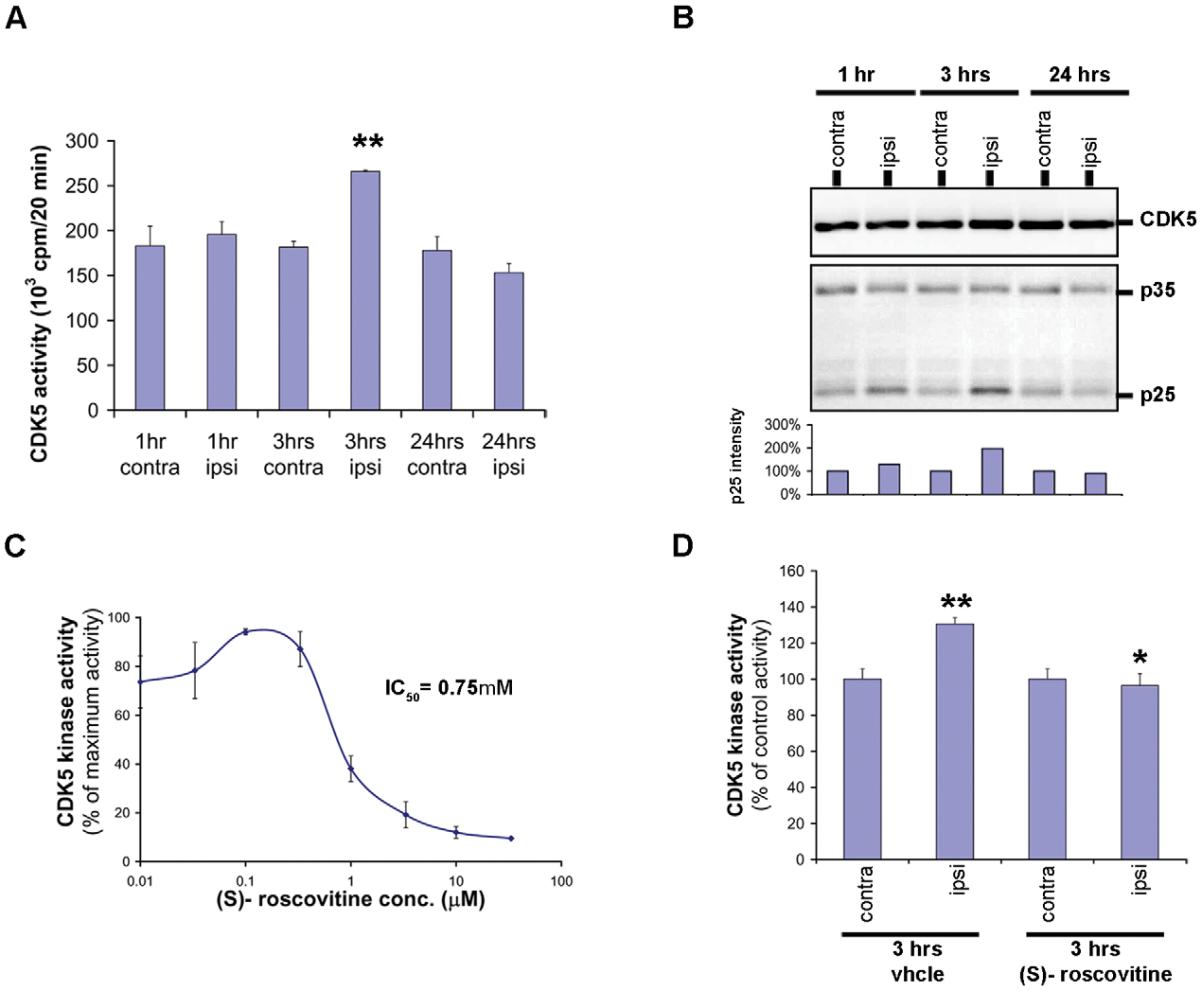
(S)-roscovitine inhibits the increase of CDK5 activity in 3 hrs pMCAo mouse brain. (**A**) CDK5 activity was evaluated in total mouse brain extracts after 1, 3, or 24 hrs of pMCAo. Both contra- (non-ischemic) and ipsi- (ischemic) lateral hemispheres were analyzed. CDK5 activity was assayed after purification by immunoprecipitation as described in the material and methods section. CDK5 activity was significantly increased in the ipsi-lateral hemisphere 3 hrs after the occlusion. (**B**) The amount of active CDK5/p35 or p25 complexes was estimated after purification on (S)-roscovitine beads (500 µg/chromatography) by SDS-PAGE followed by Western blotting using anti-CDK5 and anti-p35/p25 antibodies. The p25 band intensity in the ipsi (ischemic)-lateral hemispheres is increased after occlusion with a maximum at 3 hrs (values correspond to the percentage of control intensity and were normalized using the CDK5 band intensity which remained constant). (**C**) CDK5 activity purified from mouse brain was inhibited by (S)-roscovitine with an IC_50_ of 0.75 µM. (**D**) The increase of CDK5 activity in 3 hrs post-pMCAo brain was prevented by *in vivo* treatment with (S)-roscovitine. Three different mice were treated by IP injection of (S)-roscovitine and sacrificed 3 hrs post-pMCAo. The significant difference between CDK5 activities detected in contra- and ipsi-lateral hemispheres of vehicle control treated animals was not observed when the animals received (S)-roscovitine treatment. ** p<0.05, *t*-test.

CDK5 activity was next evaluated in our permanent mouse stroke model. Our data revealed a significant transient increase of CDK5 activity in the lesioned ipsilateral hemisphere 3 hrs after the onset of pMCAo, compared to CDK5 activity of the control contralateral hemisphere (**Figure 4A**). At this time point, the level of p25 protein was also increased in the ischemic hemisphere, compared to that of the control hemisphere (**Figure 4B**). These data suggested that pMCAo induced cleavage of p35 to p25, with subsequent activation of CDK5/p25 activity. This activation was detectable as early as 1 hr and as late as 6 hrs post-pMCAo (data not shown). Ischemic mouse brain CDK5 activity was confirmed to be inhibited by (S)-roscovitine (IC_50_ of 0.75 µM) (**Figure 4C**). CDK5 activity level was then evaluated in 3 hrs post-pMCAo brains of animals after systemic administration of (S)-roscovitine. Our data revealed that the transient increase of CDK5 activity previously observed in the ischemic ipsilateral brain hemisphere was significantly prevented by *in vivo* treatment with (S)-roscovitine (**Figure 4D**).

## Discussion

Our data shows that the (S) stereoisomer of roscovitine, a CDK inhibitor, exerts a neuroprotective effect *in vivo*, after systemic pre- and post-ischemia administration in experimental stroke models. Furthermore, we show that this *in vivo* neuroprotection is mediated in part by inhibition of CDK5.

It is now accepted that the (R) stereoisomer of roscovitine displays consistent neuroprotective efficacy in both *in vitro* and *in vivo* models of neuronal death [22], [32]–[34]. Only one stroke study was however recently reported using a transient model of cerebral ischemia in rat [22]. In this study, drug was administered in prevention and through an ICV delivery mode that are not suitable for stroke patient therapy. Our data revealed that the neuroprotective effect mediated by systemic administration (IP) of (S)-roscovitine, the levogyre form of roscovitine, is similar to that obtained after ICV administration, showing that there is no loss of neuroprotective effect through systemic administration. Moreover, we showed that (S)-roscovitine was able to cross brain barrier, in healthy animals, as already shown for the (R)-roscovitine [24], [25]. (S)-roscovitine concentration in the brain was 15% of that observed in the plasma. Even if it is possible that ruptured BBB in ischemic animals increased brain roscovitine concentration, our data suggested that such a concentration fraction was sufficient to obtain a beneficial effect after stroke. Furthermore, we developed an injectable formulation of (S)-roscovitine that is suitable for human therapy. Systemic use of (S)-roscovitine solubilized in cyclodextrin excipient showed its neuroprotective effect in a transient model of focal ischemia when the drug is administered not only prior to the ischemic insult, but also after a 2 hrs time delayed administration. Furthermore, we showed that the lower tested (S)-roscovitine doses exhibited stronger neuroprotective effects than the high dose, suggesting that (S)-roscovitine displayed a biphasic U-shaped dose-response relationship as frequently observed in neuroprotection studies [35].

How does (S)-roscovitine protect neurons from excitotoxic death and ischemic damage? Kinase-inactive roscovitine derivatives (N^6^-methyl-(R)-roscovitine and O^6^-(R)-roscovitine) displayed no significant neuroprotective effect, strongly suggesting that (S)-roscovitine neuroprotective effect is CDK- mediated. CDK5 has been identified as a putative therapeutic target in ischemia [11]. We confirmed that CDK5/p25 activity is increased in the ischemic cerebral tissue as previously described [19], [21], [36]. Our *in vivo* data revealed that this increase in CDK5 activity was prevented by the systemic administration of (S)-roscovitine, supporting the hypothesis that CDK5 is involved in its neuroprotective effects. It has been shown that (R)-roscovitine reduced hyperphosporylation of the Tau protein, a major CDK5 target, after transient cerebral ischemia in rat, mainly by inhibiting CDK5 [21]. Although CDK5 is undoubtedly involved in neuroprotection, it may not be the only relevant target. (S)-roscovitine indeed potently inhibits CDK1, CDK2, CDK7, CDK9 [37], as well as Erk1/Erk2 and CK1 [30], that play key roles in neuronal development and survival [38]. Increasing evidence indisputably links cell cycle CDKs to the pathogenesis of stroke [10], [11]. Intracerebral administration of the CDK inhibitor flavopiridol significantly reduced ischemic damage during focal and global stroke [39], [40]. Although these studies focused on the inhibition by flavopiridol of the cell cycle CDKs (CDK4 and CDK6), it is important to notice that this compound also displays a strong inhibition of CDK5 [18], [41]. Therefore, a combined inhibition of CDK5 and other kinase targets by (S)-roscovitine may be essential to provide neuroprotection in ischemic tissue.

Is (S)-roscovitine a suitable potential therapeutic agent for stroke? Because of a slightly better activity of (R)-roscovitine on CDKs compared to the (S)- isomer [30], [37], the (R) stereoisomer has been most frequently chosen in basic research studies. Furthermore it has been extensively investigated as a potential anti-cancer drug (under the names of CYC202 or Seliciclib). It is currently in late phase 2 clinical trials against non small cell lung cancer and nasopharyngeal cancer [42]. Despite this fact, we observed however that (S)-roscovitine displays a better neuroprotective efficacy than the (R) isomer in our culture system. (S)-roscovitine displays significant neuroprotective efficacy in both permanent and transient focal ischemia models in rodent. Besides, (S)-roscovitine significantly decreased the total infarct volume when administered after the ischemia onset, although the precise therapeutic time window still needs to be studied. Finally, we show that (S)-roscovitine neuroprotective efficacy is observed following not only ICV but also systemic administration. More recently, it has been shown that (R)-roscovitine attenuates cell cycle activation in glial cells, therefore reducing glial scar formation and microglial activation [22], [23]. Although anti-inflammatory properties of the (S) stereoisomer of roscovitine remain to be demonstrated, all these observations strongly support a multimodal action for these compounds.

In conclusion, our results strongly support the idea that systemic administration of (S)-roscovitine reduces brain damage after cerebral ischemia in part through a CDK5 mechanism. Roscovitine acts, on different cell types (neurons and glia) and through various mechanisms: anti-apoptotic, anti-excitotoxic, and possibly anti-inflammatory pathways. Our study revealed that (S)-roscovitine successfully meets 7 over the 10 criteria recommended by the STAIR criteria [8]. Complementary preclinical studies therefore need still to be addressed, such as long term studies of (S)-roscovitine neuroprotective effect, evaluation of its beneficial activity on animal behavior, and association with thrombolytic agent such as rt-PA. Our findings provide however encouraging support for the further development of CDK inhibitors as therapeutic drugs to reduce the neurodegeneration associated with stroke.

## Materials and Methods

### Drugs

**Figure 5:**
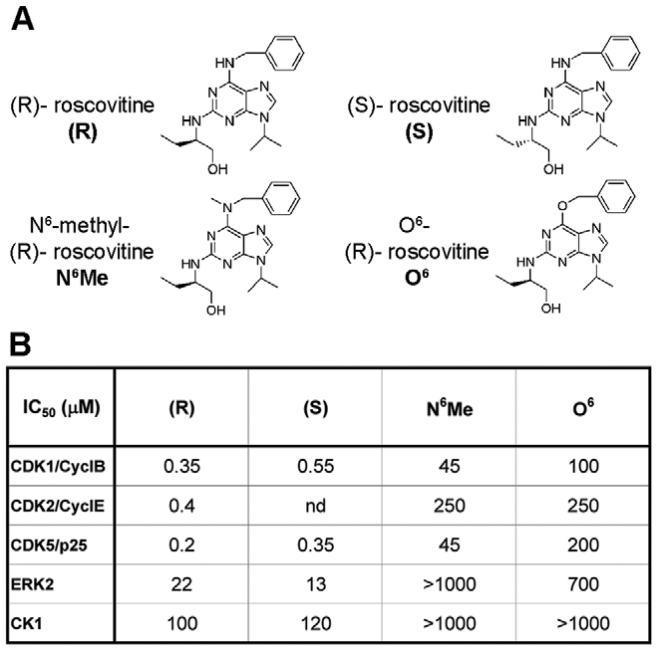
Structure of roscovitine analogs used in this study. (**A**) Structure of (R)- and (S)- stereoisomer of roscovitine, as well as N^6^-methyl (R)-roscovitine and O^6^-(R)-roscovitine that are two compounds designed for their inability to inhibit CDKs [30] (**B**) IC_50_ values (in µM) previously described for these compounds [30], [31]. nd: not determined.

Both (R) and (S) stereoisomers of roscovitine were studied in this study (**Figure 5A, B**) [37]. Two compounds designed for their inability to inhibit CDKs, N^6^-methyl (R)-roscovitine and O^6^-(R)-roscovitine, were also used (**Figure 5A, B**) [30].

### Animals

P60 male C57 b/6 mice (20–25 g; Janvier Breeding Center), P60 male Sprague Dawley (SD) rats (280–320 g; Janvier Breeding Center or Harlan Inc) were housed under controlled standard conditions (diurnal lighting conditions with food and water available *ad libitum*). Mice were fasted the day before the surgery. Animal experimentation performed at Neurokin (pMCAo on C57b/6 mice and tMCAo on SD rats) was conducted in respect to the European Communities Council Directive (86/609/EEC), to the French Department of Agriculture (license n^o^ 13.343 to BM), and to the local Veterinary Authorities (license n^o^ A 13 055 19 to Institut de Neurobiologie de la Méditerranée). No ethical committee approval was requested for these experiments because such authorization is not required in France. Animal experimentation performed at MDS Pharma Services (tMCAo on SD rats) was conducted in compliance with the study protocol, current MDS PS SOPs and adhered to regulations outlined in the USDA Animal Welfare Act Act 9 CFR Parts 1–3 (Federal Register 39129, July 22, 1993) and the conditions specified in the Guide for the Care and Use of Laboratory Animals (ILAR Publication 1996, National Academy Press). This protocol was reviewed and approved by MDS Pharma Services' Institutional Animal Care and Use Committee (IACUC).

IACUC PRF Number: S0001 03-25-07

IACUC Protocol Amendment #: S08153 (AA76553)

IACUC Approval date: 20-Aug-2008

IACUC Protocol Amendment #: S08153 (AA76553), Amendment 2

IACUC Approval date: 09-Dec-2008

No alternative test systems were identified as replacement for the use of live animals in all these *in vivo* studies.

### Injectable (S)-roscovitine formulation

The solubilization of (S)-roscovitine was studied using different surfactants commercially available for human injectable formulation. These excipients were Polysorbate 80 (Tween 80; Merck), Cremophor EL (BASF), and hydroxypropyl β-cyclodextrin (HPbCD; Sigma). Briefly, an excess amount of (S)-roscovitine was shaken in a precise volume of each excipient for 24 hrs at 20°C. The supernatant was isolated and the quantity of solubilized (S)-roscovitine analyzed by HPLC, according to Drugabilis S.A.R.L. (Chatenay Malabry, France) procedures.

### Blood brain barrier permeability

SD adult rats received intravenous injection of (S)-roscovitine solution (HPbCD 30% pH 7.4) at 2 doses (20 and 30 mg/kg; n = 2 rats/dose). Dosed animals were sacrificed at 15 min after the end of the intravenous injection. Blood were collected through the retro-orbital sinus using a capillary tube. Animals were then perfused with saline solution into the heart to extract the maximum blood sample from the brain. Brain was collected, and homogenized. Both plasma and brain samples were analyzed using LC/MS/MS determination, according to ADME-Bioanalysis (Vergeze, France) procedures.

### Permanent middle cerebral artery occlusion, pump implantation and drug administration in mice

Permanent occlusion of the middle cerebral artery (pMCAo) was performed on P60 male C57 b/6 mice according to the previously described protocol [26] modified from [27]. Briefly, animals were anesthetized intraperitoneally with chloral hydrate (500 mg/kg). Coagulation of the left MCA was performed with a bipolar diathermy. During the surgical procedures, animals were maintained at 37°C using a temperature retro-controlled heating pad (Harvard Apparatus) and under a heating lamp until full recovery. Animal body temperature and glucose concentration of tail vein blood were recorded 3 times during the surgery: prior to surgery, immediately after the occlusion and at 3 hrs post-occlusion right before the sacrifice. Animals were sacrificed 1, 3, and 24 hrs after the occlusion either by cervical dislocation or by intracardiac perfusion with 4% PFA.

Scheme of the experimental procedures is shown in **Figure 1A**. For intracerebroventricular administration of drug, (S)-roscovitine at a concentration of 50 mM in DMSO was diluted to 500 µM into Krebs-Ringer's solution (pH 7.4) containing 0.01% Evans blue. Drug (n = 5 mice) or its vehicle control (n = 5 mice) were administrated to the animals through infusion into the lateral ventricle (coordinates antero-posterior = 0.0, lateral = −0.8, depth = 2.2; according to Bregma) using micro-osmotic pumps (1 µl/hr; Alzet model 1003D) starting 48 hrs before the occlusion. For intraperitoneal (IP) administration mode, (S)-roscovitine at an initial concentration of 10 mM in 0.05 M HCl were diluted in 0.05 M HCl to 25 mg/kg (pH 1.5). 0.05 M HCl was used for the vehicle-treated animals. Drug (n = 5 mice) or its vehicle control (n = 5 mice) were administrated to the animals through IP injection (250 µl) at 15 min before and 1 hr after pMCAo.

### Transient middle cerebral artery occlusion (tMCAo) and drug administration in rats

Animals were anesthetized with gaseous isofluorane. Body temperature was monitored with a rectal temperature probe and maintained with a heating pad at approximately 37°C throughout experiment. An incision was made in the scalp and the right temporal-lateral portion of the skull was exposed. A miniature plastic probe holder was then fixed to the skull with Super Glue to allow placement of a laser-Doppler probe directly over a region of the cortex supplied by the middle cerebral artery. CBF was continuously recorded by Laser Doppler flowmetry during a period covering induction of cerebral ischemia (from 8 to 12 min before- to 10 min after- the occlusion). The mean CBF value was calculated before surgery (a control period lasting 10 min) and after MCAo (an ischemic period lasting 5 min after the occlusion). The percentage of CBF drop was calculated as follow: [1-(mean CBF during ischemic period/mean CBF during control period)] x 100.

Transient focal cerebral ischemia (90 or 120 min) was performed as previously described [43]. Briefly, the right common carotid artery (CCA), the internal carotid artery (ICA), and the external carotid artery (ECA) were exposed through a midline incision of the neck. The pterygopalatine artery was occluded. A 5.0 prolene filament suture, treated with a glue coating to enlarge the diameter tip (375 µm) was used as an occluder. The occluder was inserted through a small incision into the ECA and was gently advanced into the ICA, until a drop in CBF of 75% or greater associated with a slight thread progression resistance was observed. Animals that did not show a CBF drop of 75% or greater at this point were immediately removed from the study and humanely euthanized. Rats recovered from anesthesia and returned to their home cages. Ninety minutes (Neurokin study) or 120 min (MDS PS study) later, rats were re-anesthetized and the occluder removed to allow reperfusion. Animal body temperature and glucose concentration of tail vein blood were measured 3 times during the surgery: prior to surgery, immediately after the occlusion, and immediately after reperfusion. Animals were sacrificed either at 48 hrs (Neurokin study) or 72 hrs (MDS PS study) after occlusion by cervical dislocation.

Schemes of the experimental procedures are shown in **Figures 2A, C**. For experiments done at Neurokin (**Figure 2A**), (S)-roscovitine (n = 16 rats) or vehicle (n = 18 rats) was injected into the animals through a IV bolus (25 mg/kg in HPbCD 30%, pH 7.4) followed by 3 successive SC injections (54 mg/kg in saline HCl 50 mM, pH 1.5) performed 15 min before and at 24 and 29 hrs post-occlusion. For experiments performed at MDS Pharma Services (**Figure 2C**), drug (n = 11–12 rats) or vehicle (n = 13 rats) was administered through a bolus IV (25 mg/kg in HPbCD 30%, pH 7.4) followed by immediate SC infusion for 48 hrs, and performed either at 15 min prior or 2 hrs 15 min after the occlusion. Several drug doses were tested for the SC infusion (10, 5, and 1 mg/kg in saline HCl 50 mM, pH 1.5). Animal treatments were performed in a blind manner in both tMCAo studies.

### 2, 3, 5-triphenyl tetrazolium chloride (TTC) staining and evaluation of neuronal death

One or two mm-thick coronal sections were prepared from freshly dissected brains, stained at 37°C in solution of 1% 2,3,5-TTC (Sigma) in 0.9% saline for 10 min, and fixed overnight with 4% PFA. To measure brain infarct, images from the stained coronal sections were captured using a digital camera and analyzed with the ImageJ (NIH) image processing software. In pMCAo mouse brains, both core and hypometabolic zone were delineated using the ImageJ free-hand tool for every 1 mm thick-section, and their volumes determined after calibration using the software. Total area volumes were determined as the addition of the volume of both core and hypometabolic zone. In tMCAo rat brains, total, cortical, and subcortical volumes of the lesion were calculated on 2 mm thick- TTC stained sections either using the ImageJ software as previously described or a computerized image analysis system (Simple PCI; Compix Inc.). The infarct volumes were captured as actual volumes, and then corrected to compensate for the presence of brain swelling in the infarcted hemisphere. A correction factor was calculated by dividing the volume of the contralateral hemisphere by the ipsilateral hemisphere. Infarct volumes were then multiplied by this correction factor to obtain edema- corrected infarct volumes.

### E18 mixed hippocampal cells, pharmacological treatments and evaluation of neuronal death

Mixed hippocampal cell cultures were prepared from embryonic day 18 (E18) Wistar rats as previously described [44] and grown *in vitro* for 10 days. Treatments were done by directly adding KA (200 µM; Sigma), drugs, and/or vehicle (DMSO 0.1%) to the medium and left for 5 hrs. No significant KA-induced toxic effect was ever detected on glial cells at this concentration (date not shown). The effects of roscovitine compounds [(R)-, (S)-, N^6^-methyl-(R)-, or O^6^-(R)-roscovitine] were determined by adding different concentrations of the molecules ranging from 0.05 to 50 µM to the medium at the same time as the KA addition. The cell death marker, propidium iodide (PI; 7.5 µM; Sigma), was added to the medium at 4 hrs. Neuronal death was evaluated at 5 hrs by combining phase contrast and fluorescent microscopy observations. Neurons from random and representative fields were counted at low magnification (4x). At least 5 fields per condition (number total of neurons exceeding in general 150) were examined from 3 independent cultures. For every condition in every experiment, percentage of neuronal death was expressed as the ratio between PI-positive neurons and the total number of neurons visualized by phase contrast microscopy. To assess neuroprotection, relative neuronal death (RND) was calculated in 3 independent cultures and a neuroprotection index (NI) defined as: RND = (% of neuronal death with KA/roscovitine – % of neuronal death with roscovitine)/(% of neuronal death with KA - % of neuronal death with vehicle), and NI = 1-RND. By definition, relative percentage of neuronal death (RND) in KA-treated culture was 100% and neuroprotection index (NI) was 0%.

### Affinity chromatography on immobilized drugs

The preparation of N^6^-methyl-(R)-roscovitine and (R) - and (S)-roscovitine resins was described previously [30], [31]. Healthy or pMCAo mouse brains were freshly dissected, ipsi (ischemic) - and contra (non-ischemic) - lateral hemispheres isolated and snap frozen until further use. Tissues were homogenized and sonicated in homogenization buffer (PY buffer: 60 mM β-glycerophosphate, 15 mM p-nitrophenylphosphate, 25 mM Mops (pH 7.2), 15 mM EGTA, 15 mM MgCl_2_, 1 mM DTT, 1 mM Na vanadate, 1 mM NaF, 1 mM phenylphosphate, 10 µg leupeptin/ml, 10 µg aprotinin/ml, 10 µg soybean trypsin inhibitor/ml and 100 µM benzamidine). Homogenates were centrifuged for 10 min at 14,000 g at 4°C. The supernatant was recovered, assayed for protein content (Bio-Rad protein assay) and immediately loaded batch wise on the affinity matrix. Just before use, 10 µl of packed roscovitine beads were washed with 1 ml of bead buffer (50 mM Tris pH 7.4, 5 mM NaF, 250 mM NaCl, 5 mM EDTA, 5 mM EGTA, 0.1% NP-40, 10 µg/ml of leupeptin, aprotinin and soybean trypsin inhibitor and 100 µM benzamidine) and resuspended in 600 µl of the same buffer. The tissue extract supernatant (500 µg total protein) was then added; the tubes were rotated at 4°C for 30 minutes. After a brief spin at 10,000 g and removal of the supernatant, the beads were washed 4 times with bead buffer before addition of 60 µl 2X Laemmli sample buffer. Following heat denaturation for 3 min. bound proteins were analyzed by SDS-PAGE and Western blotting or silver staining.

### Electrophoresis, Western blotting, and silver staining

Following heat denaturation for 3 min, the proteins bound to the roscovitine matrix were separated by 10% SDS-PAGE followed by immunoblotting analysis or silver staining using a GE Healthcare SDS-PAGE silver staining kit. For immunoblotting, proteins were transferred to 0.45 µm nitrocellulose filters. These were blocked with 5% low fat milk in Tris-Buffered Saline - Tween-20, incubated for 1 hr with antibodies and analyzed by Enhanced Chemiluminescence (ECL, GE Healthcare). The following antibodies were used: anti-CDK5 (1∶500; Santa Cruz Biotechnology), anti-Erk1/2 (1∶4000; Sigma), anti-p35/p25 (1∶500; Santa Cruz Biotechnology), or anti-PDXK (1∶500; [30]).

### Immunoprecipitation and protein kinase assays

Brain lysates (500 µg) were incubated with 0.8 µg of p35 antibodies (Santa Cruz Biotechnology). The CDK5/p35 and CDK5/p25 immunocomplexes were washed 4 times with bead buffer (50 mM Tris pH 7.4, 5 mM NaF, 250 mM NaCl, 5 mM EDTA, 5 mM EGTA, 0.1% NP-40, 10 µg/ml of leupeptin, aprotinin and soybean trypsin inhibitor and 100 µM benzamidine) and one time with buffer C (60 mM β-glycerophosphate, 30 mM p-nitrophenylphosphate, 25 mM Mops (pH 7.0), 5 mM EGTA, 15 mM MgCl2, 1 mM DTT, 0.1 mM sodium vanadate). The CDK5 kinase activity was assessed directly on the beads in buffer C, with 1 mg histone H1/ml, in the presence of 15 µM [γ-^33^P] ATP (3,000 Ci/mmol; 10 mCi/ml) in a final volume of 30 µl. After 20 min of incubation at 30°C, 20 µl aliquots of supernatant were spotted onto 2.5×3 cm pieces of Whatman P81 phosphocellulose paper, and, 20 seconds later, the filters were washed four times (for at least 5 min each time) in a solution of 10 ml phosphoric acid/liter of water. The wet filters were counted in the presence of 1 ml ACS (GE Healthcare) scintillation fluid.

### Statistical analysis

Results are expressed as mean +/− SEM. For Gaussian distributed continuous variable two tailed (one tailed for 2 hrs tMCAo studies) paired Student's *t*-test was used. For non-Gaussian distributed continuous variables, the nonparametric Mann-Whitney *U* test was used. The alpha level was set at 0.05.

## Supporting Information

Figure S1 A hypometabolic zone is observed 3 hrs after pMCAo in the adult mouse brain. Adult C57 b/6 mice were submitted to permanent middle cerebral artery occlusion (pMCAo) and their brains were analyzed 3 hrs after the occlusion by mitochondrial-activity TTC, TUNEL or FluoroJade staining. (A) Gray scale digital pictures of TTC- stained 1 mm- thick coronal sections of an adult mouse brain 3 hrs after pMCAo. Three areas were distinguished and delineated based on their white/gray scale densities: the darker density displaying the lowest score corresponded to the healthy area, the moderate score to the hypometabolic zone while the lowest density corresponded to the core. (B) Relative white densities of the core, hypometabolic zone and healthy areas were quantified using the ImageJ software on gray-scaled digitalized pictures in 7 independent coronal sections after 3 hr of pMCAo and TTC staining. In comparison to the healthy region, the intensity increased by 73% in the hypometabolic zone and by 204% in the core area. Note the 75% increase of white density between the core and the hypometabolic zone. (C–E) Bright field (C) and confocal fluorescent (D. E) photomicrographs of 50 µm thick coronal brain sections 3 hr after pMCAo labeled either with TUNEL (C) or FluoroJade B (FJB) (D, E). Section in C was counterstained with cresyl violet. Note in C that TUNEL-positive cells were identified in the core but were absent of the hypometabolic zone. In contrast, FluoroJade B-labeled neurons were found in both core and hypometabolic zone (D, E). Scale bars: A: 5 mm; C: 100 µm; D, E: 130 µm *, #p<0.01 t-test.
(0.56 MB TIF)

Figure S2 Specific neuronal death induced by excitotoxic KA on mixed hippocampal cultures. (A–C) Hippocampal cells isolated from E18 rat embryos and grown in vitro for 10 or 15 days (div) were characterized by immunocytochemistry with cell type specific antibodies and patch clamp recording. (A) Bright field photomicrograph of the 10 div cell culture by phase contrast. (B) Fluorescence confocal photomicrograph of the 10 div culture labeled with GFAP- (red), beta III tubulin- (green), and O4- (blue) antibodies. Hippocampal cultures contained both neuronal and glial cell types. (C) Traces showing voltage clamp recording in whole-cell configuration of neurons grown for 10 (top trace) and 15 (bottom trace) div. Note that 15 div neurons display large and frequent postsynaptic currents, reflecting a more mature neuronal network at the latest in vitro stage. (D) A model of neuronal excitotoxicity was developed using 10 div mixed hippocampal cultures and KA. Fluorescence photomicrographs of cultures exposed to either vehicle control (left panel) or 200 µM KA (right panel) and labeled with the neuronal anti-beta III tubulin antibody (top panel) or the cell death marker PI (bottom panel). Note a decrease in the density of beta-tubulin-positive neurons and an increase of that of PI-labeled cells in the KA-treated cultures in comparison to the vehicle-treated cultures. (E) Relative percentage of beta III tubulin-positive cells in the culture after vehicle- or KA- treatment. Note about 40% decrease in the neuronal density in the culture after KA exposure. (F) Dose-dependent response of neuronal excitotoxicity after a 5 hrs exposure to either vehicle or different KA concentrations ranging from 20 to 400 µM. In our cell culture model, treatment with 200 µM KA for 5 hrs was necessary to obtain approximately 50% of neuronal loss. Scale bars: A: 400 µm, B: 475 µm, D: 900 µm * p<0.01 t-test.
(0.80 MB TIF)

Table S1 Table summarizing the physiological parameters of the pMCAo C57 b/6 mice measured at different time points of the surgical procedures.
(0.03 MB DOC)

Table S2 Table summarizing the physiological parameters of the tMCAo SD rats (Neurokin study) measured at different time points of the surgical procedures. ** p<0.05, * p<0.01, t-test.
(0.03 MB DOC)

Table S3 Table summarizing the physiological parameters of the tMCAo SD rats (MDS PS study) measured at different time points of the surgical procedures. ** p<0.05, t-test.
(0.04 MB DOC)
