# Supplementary figures and images for: Delayed Treatment with Systemic (S)-Roscovitine Provides Neuroprotection and Inhibits In Vivo CDK5 Activity Increase in Animal Stroke Models
Source: PLoS One. 2010 Aug 12;5(8):e12117. doi: 10.1371/journal.pone.0012117 (PMC2920814; doi:10.1371/journal.pone.0012117)

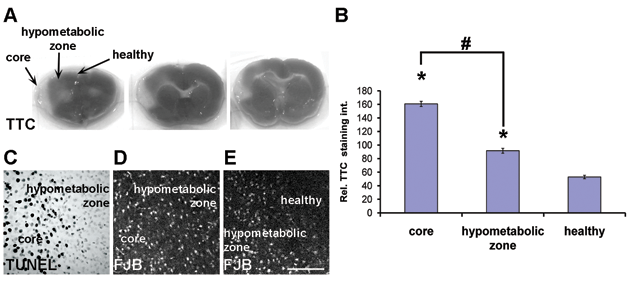

Supplement: Figure S1 — A hypometabolic zone is observed 3 hrs after pMCAo in the adult mouse brain. Adult C57 b/6 mice were submitted to permanent middle cerebral artery occlusion (pMCAo) and their brains were analyzed 3 hrs after the occlusion by mitochondrial-activity TTC, TUNEL or FluoroJade staining. (A) Gray scale digital pictures of TTC- stained 1 mm- thick coronal sections of an adult mouse brain 3 hrs after pMCAo. Three areas were distinguished and delineated based on their white/gray scale densities: the darker density displaying the lowest score corresponded to the healthy area, the moderate score to the hypometabolic zone while the lowest density corresponded to the core. (B) Relative white densities of the core, hypometabolic zone and healthy areas were quantified using the ImageJ software on gray-scaled digitalized pictures in 7 independent coronal sections after 3 hr of pMCAo and TTC staining. In comparison to the healthy region, the intensity increased by 73% in the hypometabolic zone and by 204% in the core area. Note the 75% increase of white density between the core and the hypometabolic zone. (C–E) Bright field (C) and confocal fluorescent (D. E) photomicrographs of 50 µm thick coronal brain sections 3 hr after pMCAo labeled either with TUNEL (C) or FluoroJade B (FJB) (D, E). Section in C was counterstained with cresyl violet. Note in C that TUNEL-positive cells were identified in the core but were absent of the hypometabolic zone. In contrast, FluoroJade B-labeled neurons were found in both core and hypometabolic zone (D, E). Scale bars: A: 5 mm; C: 100 µm; D, E: 130 µm *, #p<0.01 t-test. (0.56 MB TIF) [file pone.0012117.s001.tif]

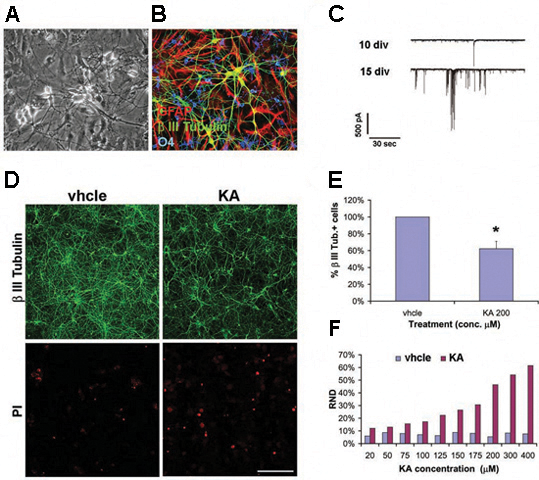

Supplement: Figure S2 — Specific neuronal death induced by excitotoxic KA on mixed hippocampal cultures. (A–C) Hippocampal cells isolated from E18 rat embryos and grown in vitro for 10 or 15 days (div) were characterized by immunocytochemistry with cell type specific antibodies and patch clamp recording. (A) Bright field photomicrograph of the 10 div cell culture by phase contrast. (B) Fluorescence confocal photomicrograph of the 10 div culture labeled with GFAP- (red), beta III tubulin- (green), and O4- (blue) antibodies. Hippocampal cultures contained both neuronal and glial cell types. (C) Traces showing voltage clamp recording in whole-cell configuration of neurons grown for 10 (top trace) and 15 (bottom trace) div. Note that 15 div neurons display large and frequent postsynaptic currents, reflecting a more mature neuronal network at the latest in vitro stage. (D) A model of neuronal excitotoxicity was developed using 10 div mixed hippocampal cultures and KA. Fluorescence photomicrographs of cultures exposed to either vehicle control (left panel) or 200 µM KA (right panel) and labeled with the neuronal anti-beta III tubulin antibody (top panel) or the cell death marker PI (bottom panel). Note a decrease in the density of beta-tubulin-positive neurons and an increase of that of PI-labeled cells in the KA-treated cultures in comparison to the vehicle-treated cultures. (E) Relative percentage of beta III tubulin-positive cells in the culture after vehicle- or KA- treatment. Note about 40% decrease in the neuronal density in the culture after KA exposure. (F) Dose-dependent response of neuronal excitotoxicity after a 5 hrs exposure to either vehicle or different KA concentrations ranging from 20 to 400 µM. In our cell culture model, treatment with 200 µM KA for 5 hrs was necessary to obtain approximately 50% of neuronal loss. Scale bars: A: 400 µm, B: 475 µm, D: 900 µm * p<0.01 t-test. (0.80 MB TIF) [file pone.0012117.s002.tif]
