# Supplementary material for: Delayed Treatment with Systemic (S)-Roscovitine Provides Neuroprotection and Inhibits In Vivo CDK5 Activity Increase in Animal Stroke Models
Source: PLoS One. 2010 Aug 12;5(8):e12117. doi: 10.1371/journal.pone.0012117 (PMC2920814; doi:10.1371/journal.pone.0012117)
Supplement: Table S1 — Table summarizing the physiological parameters of the pMCAo C57 b/6 mice measured at different time points of the surgical procedures. (0.03 MB DOC) [file pone.0012117.s003.doc]

| **C57 b/6 pMCAo mice** | **parameters** | **Vehicle (IP)** | **(S)- rosco (IP)** |
| --- | --- | --- | --- |
| **Pre-occlusion** | Body temp (^o^C)  Glucose (mg/dL) | 36.9 +/- 0.0  126 +/- 11 | 36.8 +/- 0.1  138 +/- 7.3 |
| **Post-occlusion** | Body temp (^o^C) | 36.9 +/- 0.1 | 36.8 +/- 0.1 |
| **3 hrs**  **post-occlusion** | Body temp (^o^C)  Glucose (mg/dL) | 34.5 +/- 10.6  106 +/- 7 | 34.6 +/- 0.7  110 +/- 12 |
