# Supplementary material for: Delayed Treatment with Systemic (S)-Roscovitine Provides Neuroprotection and Inhibits In Vivo CDK5 Activity Increase in Animal Stroke Models
Source: PLoS One. 2010 Aug 12;5(8):e12117. doi: 10.1371/journal.pone.0012117 (PMC2920814; doi:10.1371/journal.pone.0012117)
Supplement: Table S2 — Table summarizing the physiological parameters of the tMCAo SD rats (Neurokin study) measured at different time points of the surgical procedures. ** p<0.05, * p<0.01, t-test. (0.03 MB DOC) [file pone.0012117.s004.doc]

| **SD tMCAo rats**  **(Neurokin)** | **parameters** | **vehicle** | **(S)- rosco pre-** |
| --- | --- | --- | --- |
| **Pre-occlusion** | Body temp (^o^C)  Glucose (mg/dL) | 37.1 +/- 0.1  166 +/- 9 | 37.1 +/- 0.1  154 +/- 8 |
| **Post-occlusion** | Body temp (^o^C)  Glucose (mg/dL) | 36.8 +/- 0.0  193 +/- 13 | 36.8 +/- 0.1  214 +/- 15 |
| **Post-reperfusion** | Body temp (^o^C)  Glucose (mg/dL) | 38.8 +/- 0.2  155 +/- 37 | 38.1 +/- 0.2 **  193 +/- 1 0* |
