# Supplementary material for: Delayed Treatment with Systemic (S)-Roscovitine Provides Neuroprotection and Inhibits In Vivo CDK5 Activity Increase in Animal Stroke Models
Source: PLoS One. 2010 Aug 12;5(8):e12117. doi: 10.1371/journal.pone.0012117 (PMC2920814; doi:10.1371/journal.pone.0012117)
Supplement: Table S3 — Table summarizing the physiological parameters of the tMCAo SD rats (MDS PS study) measured at different time points of the surgical procedures. ** p<0.05, t-test. (0.04 MB DOC) [file pone.0012117.s005.doc]

| **SD tMCAo rats**  **(MDS)** | **parameters** | **untreated** | **vehicle** | **(S)- rosco pre-** | **(S)- rosco post- 10mg/kg/hr** | **(S)- rosco post-**  **5mg/kg/hr** | **(S)- rosco post-**  **1mg/kg/hr** |
| --- | --- | --- | --- | --- | --- | --- | --- |
| **Pre-occlusion** | Body temp (^o^C)  Glucose (mg/dL) | 37.6 +/- 0.1  91 +/- 2 | 37.3 +/- 0.1  115 +/- 8 | 37.3 +/- 0.1  104 +/- 5 | 37.5 +/- 0.1  116 +/- 11 | 37.5 +/- 0.1  98 +/- 2 | 37.6 +/- 0.1  105 +/- 4 |
| **Post-occlusion** | Body temp (^o^C)  Glucose (mg/dL) | 37.4 +/- 0.1  280 +/- 21 | 37.3 +/- 0.1  240 +/- 20 | 37.5 +/- 0.1  297 +/- 26 | 37.2 +/- 0.0  257 +/- 24 | 37.2 +/- 0.0  259 +/- 25 | 37.3 +/- 0.1  276 +/- 19 |
| **Post-reperfusion** | Body temp (^o^C)  Glucose (mg/dL) | 38.8 +/- 0.1  154 +/- 5 | 38.5 +/- 0.2  151 +/- 10 | 38.3 +/- 0.2  198 +/- 313** | 38.6 +/- 0.2  167 +/- 9 | 38.5 +/- 0.2  172 +/- 11 | 38.3 +/- 0.1  166 +/- 6 |
